# Unsupervised Anomaly Detection on Metal Surfaces Based on Frequency Domain Information Fusion

**DOI:** 10.3390/s25072250

**Published:** 2025-04-02

**Authors:** Wenfei Wu, Tao Tao, Jinsheng Xiao, Yichu Yao, Jianfeng Yang

**Affiliations:** 1 School of Electronic Information, Wuhan University, Wuhan 430072, China; wuwenfei@whu.edu.cn (W.W.); tt1295@whu.edu.cn (T.T.); xiaojs@whu.edu.cn (J.X.); 2 School of Computer Science, Northwestern Polytechnical University, Xi’an 710129, China; yichu_yao@mail.nwpu.edu.cn

**Keywords:** defect detection, unsupervised learning, frequency domain, feature fusion

## Abstract

Metal products are widely used in industrial manufacturing, and the quality of metal products is becoming more and more demanding. At present, although there are many methods for detecting defects on metal surfaces, there are still various limitations. The limited number of defect samples, unpredictable defect characteristics, and the interference of metal grain bring great challenges to metal surface defect detection. For this reason, this paper proposes an unsupervised algorithm, FFnet, based on the fusion of frequency domain information, which introduces the frequency domain features into the unsupervised detection. A method of the adaptive enhancement of features in the frequency domain is proposed to make the features on the frequency domain more concerned with anomalies rather than textures. A scale-adaptive feature reconstruction module is used to effectively fuse the spatial and frequency domain features to fully utilize the information from different domains. In addition, a feature selection module is designed to improve the anomaly detection capability and reduce the computational redundancy by selecting the most representative subset of features. The proposed method outperforms other state-of-the-art methods on the connecting rod surface image dataset. In addition, in the generalization experiments of Kolektor Surface-Defect Dataset 2, our method also achieves optimal results and demonstrates strong generalization ability.

## 1. Introduction

Defect detection has long been a closely observed topic in both industrial production and scientific research. As widely used industrial products, metallic materials play a crucial role in various fields such as manufacturing, aerospace, and the automotive industry. The surface of metallic materials often contains various types of defects, such as scratches, cracks, corrosion, and holes. These defects can significantly affect the strength, durability, and safety of the products. Therefore, the timely and accurate detection of surface defects in metals is of great practical significance for improving product quality, reducing production costs, and ensuring safety.

In traditional production settings, defect detection has often been manually performed. This method is not only inefficient but also highly subjective, making it difficult to standardize. With the development of computer technology and image processing, defect detection has gradually shifted from traditional manual inspection to automated methods based on computer vision and deep learning, especially with the application of deep learning techniques, which have significantly improved the accuracy and efficiency of defect detection.

However, metal surface defect detection technology still faces several challenges. Traditional image processing methods rely on hand-crafted features, which are susceptible to variations in lighting, surface contamination, and changes in viewing angles, leading to unstable detection results. Additionally, supervised learning-based methods [1,2] often require large annotated datasets. In the case of metal surface defect detection, the types and manifestations of defects are complex and varied. Defects often exhibit randomness and scarcity, and they may not appear in all samples. As a result, traditional supervised learning methods require a vast amount of annotated data to cover all possible defect types and scenarios, while acquiring defect samples in actual production environments is extremely difficult, leading to significant challenges when constructing datasets.

In recent years, with the advancement of deep learning technologies, an increasing number of unsupervised learning methods, such as autoencoders [3,4,5,6] and generative adversarial networks (GANs) [7,8,9], have been widely applied in anomaly detection. Unsupervised learning methods identify defects by learning the characteristics of normal samples and then detecting discrepancies between the sample to be inspected and the normal sample. These methods not only reduce the reliance on large annotated datasets but also enhance the robustness and generalization ability of the detection. However, the surface of metallic products differs from general industrial products in that it often contains complex and highly random metallic textures, and some defects may be concealed by these textures, causing significant interference in detection. Furthermore, metal surface defects typically appear in very subtle ways, making it difficult for existing detection models to effectively distinguish normal features from anomalous ones.

This study aims to analyze the challenges and difficulties associated with unsupervised anomaly detection algorithms in practical applications. Given the characteristics of metallic products, which feature random textures and subtle defects, we propose FFnet, an unsupervised anomaly detection method designed to address these challenges. FFnet is a novel framework that integrates both spatial and frequency domain features. The input image is passed through two feature extraction branches: The spatial branch utilizes the feature extraction capability of a large-scale pre-trained CNN to capture spatial information, while the frequency branch uses a patch-based discrete cosine transform (DCT) to extract information from the frequency domain. We have designed a frequency feature enhancement module (FFE) to fully leverage frequency information. Additionally, to fuse features from different domains, we have also designed two modules: the scale-adaptive feature reconstruction (SAFR) and reconstruction feature election (RFS) modules. SAFR aligns and reconstructs features from different scales, while RFS selects the most representative subset of features from the reconstructed ones. They effectively integrate features from different domains, enhancing the network performance while reducing redundant features, thus ensuring the network remains lightweight. The main contributions of this paper are as follows:

1. In this paper, we propose FFnet, a new framework for unsupervised anomaly detection that combines features in the spatial and frequency domains.
2. A frequency domain feature enhancement (FFE) method is designed to maximize the utilization of frequency domain information, thereby improving the detection performance of metal surface defects.
3. We also designed two additional modules: scale-adaptive feature reconstruction (SAFR) and reconstruction feature selection (RFS). SAFR and RFS effectively integrate features from different domains, enhancing network performance while reducing redundant features, thereby ensuring the network remains lightweight.

## 2. Related Works

### 2.1. Unsupervised Anomaly Detection

Currently, the problem of anomaly detection is mainly addressed using unsupervised and semi-supervised methods [10]. In the field of image anomaly detection, the prevailing approaches are still based on unsupervised algorithms, which can be broadly categorized into the following three types: image reconstruction-based methods, deep feature embedding-based methods and anomaly simulation-based methods.

**Reconstruction-based approaches** focus on anomaly detection and localization by training a model that uses only normal images and exploiting the inability of the module to effectively reconstruct anomalous regions. Common reconstruction techniques include the autoencoder (AE) [3,4,5,6], generative adversarial network (GAN) [7,8,9], transformer [11,12], and diffusion model [13], etc. Matsubara et al. [4] firstly introduced the variational auto-encoder (VAE) into the field of industrial anomaly detection. Kozamernik et al. [14] proposed a model for the visual quality control of KTL coatings based on VAE. This method successfully detects anomalous regions containing surface defects by calculating the negative log-likelihood of the return distribution of the decoder, and Schlegl et al. [7] were the first to apply generative adversarial networks (GANs) to anomaly localization. Balzategui et al. [15] employed a GAN-based anomaly location (AL) method to conduct quality inspection of monocrystalline solar cells. Hou et al. [16] utilized the method to construct a segmentation and assembly framework for anomaly localization. Recent studies [17,18,19] have improved the performance of anomaly detection by pre-training CNN models and reconstructing image features at multiple scales. However, in the case of complex image texture or structure, the anomalous regions may share the same features with normal regions, which leads to the anomalies being incorrectly reproduced in the image reconstruction process, thus affecting the detection results.

**Embedding-based approaches** obtain the deeply embedded features of an image through feature extraction and then generate an anomaly score map by comparing the differences between normal and test features. Typical methods [20,21,22] utilize pre-trained networks on ImageNet [23] for feature extraction. Defard et al. [20] represent the embedding of extracted anomalous patch features through multivariate Gaussian distribution. Roth et al. [21] adopt a core subset by means of a greedy strategy to form a memory bank for anomaly detection. Bae [22] et al., on the other hand, considered patch location and neighborhood relationship and used location information by constructing histograms of representative features at each location. In the anomaly detection stage, the input features are usually scored using either the Mahalanobis distance or the maximum feature distance. Embedding-based methods have achieved remarkable results in the field of anomaly detection; however, since these methods rely on pre-trained models of ImageNet for feature extraction, the embedded features are inevitably affected by the bias of the ImageNet dataset, which may affect the effectiveness of anomaly detection in specific tasks.

**Anomaly Simulation-Based approaches** simulate anomalous data by artificially synthesizing anomalies on normal images. Vitjan Zavrtanik et al. [24] proposed an end-to-end network, DRAEM, which learns and detects anomalies just synthesized from distribution patterns through discriminative training. CutPaste [25] proposed a simple strategy to generate synthetic anomalies for anomaly detection by cutting image chunks and pasting them to random locations in a large image. Simplenet [26] proposed a method of directly adding noise to the feature dimensions to simulate anomalies. In [27], a new end-to-end memory-assisted segmentation network, Memseg, is proposed to simulate anomaly samples by generating anomaly masks combined with DTD texture data [28], while RealNet [29] simulates anomalies using a diffusion model, adding interference during the diffusion process to make the synthesized anomaly images closer to actual anomalies. Although the anomaly simulation methods are effective to a certain extent, they rely too much on the quality of anomaly simulation, and therefore, there are still some limitations and challenges for unpredictable or rare types of anomalies.

### 2.2. Deep Learning in Frequency Domain

Frequency analysis has long been a powerful tool in the field of signal processing, and in recent years, its application in deep learning has gradually gained attention. The study in [30] enhanced deep convolutional neural networks for ultrasonic concrete inspection by utilizing continuous wavelet transform and transfer learning. In the field of image processing, the frequency domain information of images is also widely integrated into convolutional neural networks. In [31], frequency domain information from images was introduced into convolutional neural networks (CNNs) through JPEG encoding. The research in [32] proposed a model transformation algorithm that converts CNN models from the spatial domain to the frequency domain. In [33], discrete cosine transform (DCT) was used to integrate frequency domain information into CNNs in the form of residuals, avoiding the complex model transformation process, and squeeze-and-excitation block (SE-Block) was used to select frequency channels. Other studies [34] designed a frequency channel attention network to further optimize the use of frequency information. Additionally, [35] introduced a learnable frequency enhancement module and aligned the RGB domain with the frequency domain to more effectively utilize frequency information.

**Figure 1:**
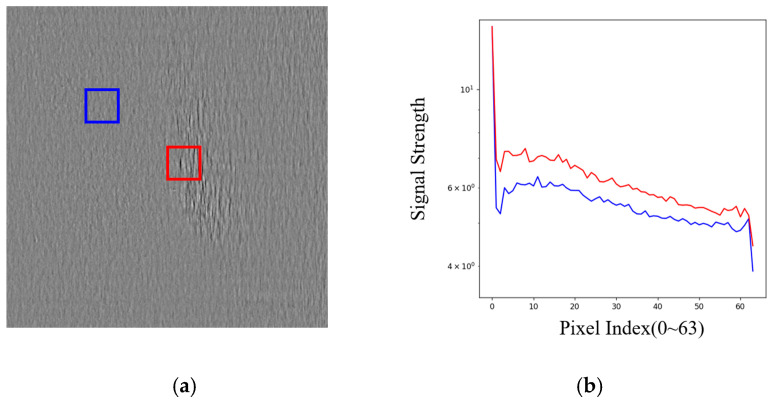
(**a**) Display of metal surface defect sample, with the abnormal region highlighted in the red box and the normal region highlighted in the blue box; (**b**) display of the frequency spectra for the two regions. The blue curve represents the frequency signal of the normal region within the blue box; the red curve represents the frequency signal of the abnormal region within the red box.

In the studies presented in [33,34,35], frequency domain information is extracted using discrete cosine transform and integrated into the CNN as residuals. These works have demonstrated the feasibility and effectiveness of fusing frequency domain features with CNN. As illustrated in the Figure 1**,** we present an example of a metallic surface defect, where the blue box indicates the normal region, and the red box highlights the defect region. Due to the presence of random metallic textures on the surface, there is a certain degree of interference with the detection of defects. We perform frequency analysis on these two regions and plot the statistical results of the frequency signal. The curve graph reveals a clear difference in the frequency signals between the two regions. Therefore, frequency information can be used as a supplement to enhance the CNN model’s ability to perceive and detect metal anomalies.

Although unsupervised anomaly detection methods have made significant progress in recent years, research on combining frequency domain and spatial domain features for anomaly detection remains scarce. Unlike existing works, we innovatively integrated both spatial and frequency domain features during feature extraction, effectively combining these two domains. Furthermore, we utilized a reconstruction module to further learn and exploit the rich information from both types of features. This cross-domain fusion approach enables the model to more effectively capture anomaly patterns in images, significantly improving detection accuracy and robustness, thus providing a more comprehensive and precise solution for anomaly detection tasks.

## 3. Proposed Method

The FFnet algorithm proposed in this paper is a novel anomaly detection framework that integrates spatial and frequency domains features. The network architecture, as shown in Figure 1, mainly consists of two feature extraction branches, a feature reconstruction module (SAFR), a feature selection module (RFS), and a discriminator.

**Figure 2:**
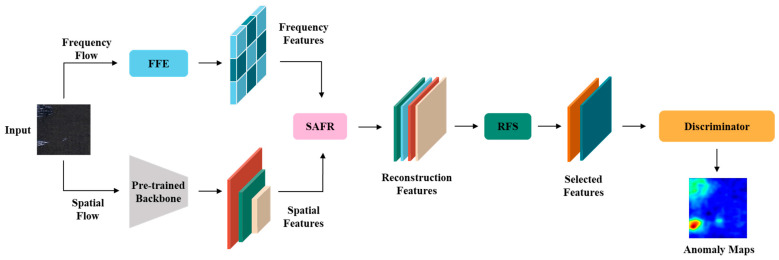
Our method employs a dual-branch feature extraction architecture that integrates both spatial and frequency information for anomaly detection.

As illustrated in Figure 2**,** our proposed method is a dual-branch feature extraction network framework that combines spatial and frequency features. The spatial feature extraction branch is similar to that of traditional methods [20,21,22], utilizing a pre-trained convolutional neural network (CNN) for feature extraction. In the frequency domain branch, patch-based discrete cosine transform is used to extract frequency domain information. To improve the applicability of the frequency domain information, we have designed a learnable frequency feature enhancement module named FFE, which adaptively enhances features across different frequency bands, allowing the network to focus more on the useful frequency bands. To effectively integrate the features from different branches, we also designed the SAFR and RFS modules. SAFR is responsible for fusing spatial and frequency features from different scales, while RFS selects the most useful channels from the reconstructed features and discards redundant ones. Finally, the anomaly discriminator, similar to previous method [26], detects anomalies by evaluating anomaly scores.

### 3.1. Frequency Domain Feature Enhancement

**Figure 3:**
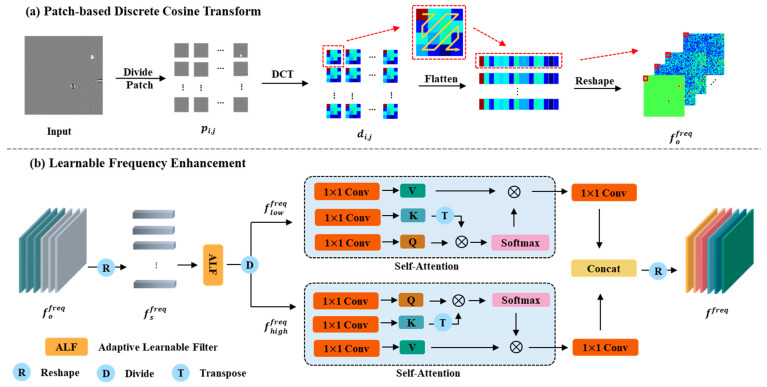
The structure of FFE consists of two steps: (**a**) patch-based DCT transformation to extract partitioned frequency domain features; (**b**) a learnable frequency feature enhancement module.

As mentioned earlier, frequency domain information can serve as a supplement to CNN, enhancing the representation of features. To better perceive and distinguish abnormal patterns, we have developed a new feature extraction framework. This framework adds an additional frequency domain feature extraction branch on top of the traditional pre-trained network feature extractor. This dual-branch feature extraction architecture enhances the representation of traditional CNN features by incorporating frequency domain information. We design a frequency feature enhancement (FFE) module to extract and enhance frequency domain features. The implementation of the FFE module primarily involves two key steps. One is a patch-based DCT transformation for extracting frequency domain features, and the other is a learnable frequency enhancement module. Its structure is shown in Figure 3.

**Patch-based Discrete Cosine Transform:** In this section, the input image needs to be transformed by DCT to obtain the frequency domain information. As shown in Figure 3a, the image is first divided into patches, with each patch having a size of k×k, resulting in a set of patches $\left\{p_{i,j}|1\leq i\leq \frac{H}{k},1\leq j\leq \frac{W}{k}\right\}$. Then, a DCT transformation is applied to each patch, converting them into frequency spectra $d_{i,j}\in R^{k\times k}$. After the DCT transformation, the frequency signals of the image are represented in the frequency spectrum, with low-frequency signals concentrated in the top-left corner and high-frequency signals in the bottom-right corner. Following the sequence shown in Figure 3a, we expand the frequency signals from high frequency to low frequency. At this point, the frequency signals of each patch are expanded into a sequence of length $k^{2}$. Next, based on the position of each patch in the original image, the frequency signals of the same frequency band are concatenated together. In this way, we transform the original image into the frequency domain, obtaining the frequency domain feature $f_{o}^{freq}\in R^{\frac{H}{k}\times \frac{W}{k}\times k^{2}}$.

The frequency domain features $f_{o}^{freq}$ obtained through the patch-based segmentation method are completely independent of each other between different patches. To enhance the correlation between neighboring patches and increase the receptive field, we apply a local feature aggregation strategy to $f_{o}^{freq}$. We define the feature vector at position $\left(h,w\right)$ in $f_{o}^{freq}$ as $f_{o}^{freq}\left(h,w\right)$, and the neighborhood of the feature vector at position $\left(h,w\right)$ as $N_{p}^{\left(h,w\right)}$. The formula is as follows:

(1) $$\begin{array}{c} N_{p}^{\left(h,w\right)}=\left\{\left(a,b\right)|a\in [h-[p/2],\ldots ,h+[p/2]],b\in [w-[p/2],\ldots ,w+[p/2]]\right\}. \end{array}$$

In this case, p represents the range of the neighborhood. We choose p=3, meaning that the neighborhood consists of the surrounding 3×3 patches. For each position $\left(h,w\right)$, we calculate the aggregated feature $f_{a}^{freq}\left(h,w\right)$ for its neighborhood $N_{p}^{\left(h,w\right)}$. The formula is as follows:

(2) $$\begin{array}{c} f_{a}^{freq}\left(h,w\right)=fagg\left(\left\{f_{o}^{freq}\left(a,b\right)|\left(a,b\right)\in N_{p}^{\left(h,w\right)}\right\}\right). \end{array}$$

In Formula (2), fagg is the function that performs the aggregation of the vectors in the neighborhood $N_{p}^{\left(h,w\right)}$. We use the adaptive mean pooling method for aggregation, and the formula is as follows:

(3) $$\begin{array}{c} fagg\left(h,w\right)=\frac{1}{p*p}\sum_{(a,b)\in N_{p}^{\left(h,w\right)}}f_{o}^{freq}\left(a,b\right). \end{array}$$

**Learnable Frequency Enhancement:** In practical industrial scenarios, we have found that the textures and defects on metal surfaces are highly complex and variable. Features derived solely from fixed DCT transforms may not meet the requirements for anomaly detection. Therefore, we designed a learnable adaptive frequency enhancement module to improve the applicability of frequency information. As shown in Figure 3b, this module consists of two main components: the adaptive learnable filter (ALF) and the self-attention mechanism (SA) [36,37]. Due to the learnable nature of ALF, it can adaptively enhance features across different frequencies. The SA mechanism can dynamically consider the mutual interactions between features across different frequencies.

First, the frequency domain features undergo a reshaping operation, where $f_{a}^{freq}\in R^{\frac{H}{k}\times \frac{W}{k}\times k^{2}}$ is reshaped into $f_{s}^{freq}\in R^{\frac{H\times W}{k^{2}}\times k^{2}}$. Define $f_{Rj}^{freq}$ as the j-th column vector of $f_{s}^{freq}$. ALF is a parameterized learnable adaptive filter with a total of H×W parameters. Let its parameters be defined as follows:

(4) $$W_{ALF}=\left\{w_{j}|w_{j}\in R^{\frac{H\times W}{k^{2}}},1\leq j\leq k^{2}\right\}.$$

Then, the $f_{s}^{freq}$ is fed into the ALF for adaptive filtering, resulting in $f_{f}^{freq}{\in R}^{\frac{H\times W}{k^{2}}\times k^{2}}$. The formula is as follows:

(5) $$f_{f}^{freq}{=F}_{ALF}\left(f_{s}^{freq}\right),$$

(6) $$F_{ALF}\left(f_{s}^{freq}\right)=\left\{w_{j}{⊙f}_{Rj}^{freq}|1\leq j\leq \frac{H\times W}{k^{2}}\right\},$$

⊙ is the element-wise multiply operation. Next, we divide the filtered features into two parts, low-frequency and high-frequency, as $f_{low}^{freq},f_{high}^{freq}{\in R}^{\frac{H\times W}{k^{2}}\times \frac{k^{2}}{2}}$. These two parts are then sent into two separate SA modules for further processing. The SA module allows features from different frequencies to interact with each other. By enhancing the low-frequency and high-frequency signals separately, we maximize the interaction between features across different frequencies while ensuring the distinction between low-frequency and high-frequency information. Finally, the outputs of the two SA modules are concatenated and reshaped back to the original dimensions, resulting in the enhanced feature $f^{freq}\in R^{\frac{H}{k}\times \frac{W}{k}\times k^{2}}$.

### 3.2. Scale-Adaptive Feature Reconstruction

**Figure 4:**
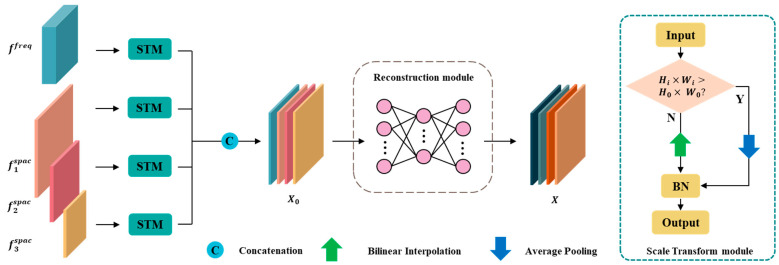
SAFR mainly consists of the STM and the reconstruction module, which are capable of fusing and reconstructing features of different sizes.

We introduce frequency domain features to assist the network in better detecting anomalies on metal surfaces. To effectively fuse frequency domain features with spatial features, we design a scale-adaptive feature reconstruction (SAFR) module. Due to the strong randomness of defects on metal surfaces, their size and shape can vary significantly. Features at different scales have varying receptive fields. Deeper features focus more on structural information, while shallower features are more sensitive to textures. Fusing features at different scales can enhance the network’s stability in dealing with scale variations. SAFR primarily consists of four scale transformation modules (STM) and a feature reconstruction module based on MLP (multi-layer perceptron), as shown in Figure 4.

In the spatial feature extraction section, we use the pre-trained WideResNet50 [38] as the backbone network. Unlike PatchCore, to retain more detailed information, we use the outputs from three layers of the backbone network as feature extractors. The extracted spatial domain features can be represented as $\left\{f_{1}^{spac},f_{2}^{spac}{,f}_{3}^{spac}\right\}$. Since spatial domain features and frequency domain features have different sizes, they need to be aligned before fusion. Here, we use the scale transformation module (STM) to perform scale normalization, ensuring that all feature blocks are adjusted to the size $\left(H_{0},W_{0}\right)$. In the STM, we use average pooling layer and bilinear interpolation to achieve the downscaling and upscaling of feature sizes. We denote the STM as $T_{s}$, and the transformation process is as follows:

(7) $$X_{i}^{spac}=T_{s}\left(f_{i}^{spac}\right),$$

(8) $$X^{freq}=T_{s}\left(f^{freq}\right).$$

The feature sizes after transformation by the STM module are listed in Table 1. Next, along the channel dimension, we concatenate the spatial features with the frequency domain features according to their corresponding positions, as shown below:

(9) $$X_{o}=Concat\left(X_{i}^{spac},X^{freq}\right).$$

At this point, the spatial and frequency domain features are in an independent state. Then, we use an MLP structure as the feature reconstruction module $G_{R}$. The MLP structure is capable of capturing complex nonlinear relationships in the data, enabling the more effective integration of features from different domains. The concatenated features $X_{o}$ are fed into the reconstruction module, producing the fused features X, which combine both spatial domain and frequency domain information, as shown below:

(10) $$X=G_{R}\left(X_{o}\right).$$

### 3.3. Reconstruction Feature Selection

**Figure 5:**
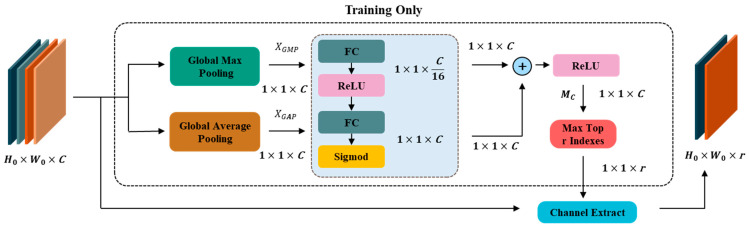
By applying global max pooling and global average pooling to the reconstructed features, we selectively extract the parts of the features that have local and global representativeness, respectively.

As mentioned in Section 3.2, to preserve more useful features, we selected the outputs of three intermediate layers in the spatial module and integrated them with frequency domain features. Not all of these features directly contribute to anomaly perception and detection. To enhance the feature representation of the model and reduce feature redundancy, we designed a reconstruction feature selection (RFS) module. Inspired by the channel attention mechanism [39], this approach directs the network to focus more on the feature information from important channels. Different channels carry varying amounts of information. Some channels contain more useful features, while others may be redundant or irrelevant. During training, a weight coefficient is assigned to each channel of the input features, where the magnitude of the coefficient indicates the importance of the corresponding channel. The channels are ranked according to their weight coefficients, retaining the most important channels while discarding the redundant ones. The RFS module selects the most representative subset of features from the reconstructed feature map in this manner, while also reducing feature redundancy, making the network more lightweight. The structure of the RFS is shown in Figure 5.

First, global maximum pooling (GMP) and global average pooling (GAP) are applied to X, yielding $X_{GMP}$ and $X_{GAP}$, respectively. Then, both are passed through a shared MLP, and the output results are summed and activated by a ReLU function to obtain a weight matrix $M_{C}$. We select the top r values from $M_{C}$ and obtain the position indexes of these r values. The channels at these r positions represent the most representative reconstructed feature channels after GMP and GAP. To ensure model consistency, the step of obtaining the r indexes through global pooling is performed only during the training process.

GMP and GAP represent local and global features in space, respectively. $X_{GMP}$ focuses more on local information, while $X_{GAP}$ emphasizes global information. By combining both, we enhance the model’s ability to perceive anomalies at different scales. At this point, we obtain the final feature representation $\chi {\in R}^{H_{0}\times W_{0}\times r}$, as shown in the following formula:

(11) $$\chi =RFS\left(X,r\right).$$

### 3.4. Discriminator and Loss Function

In the anomaly detection section, we adopt a two-layer MLP architecture as the discriminator D, which is used to assess the normality of each patch. During training, we use only normal sample images as the training set. We simulate anomalous features by adding Gaussian random noise to the normal features. We represent the normal features as $\chi_{h,w}$, where $\left(h,w\right)$ denotes the position of the patch. The simulated anomalous features are represented as $\chi_{h,w}^{-}$. $\chi_{h,w}$ represents positive samples, and $\chi_{h,w}^{-}$ represents negative samples. This anomaly simulation-based self-supervised method is used to learn the features of normal samples. The anomalous features $\chi_{h,w}^{-}$ are obtained by adding Gaussian random noise $\epsilon \in R^{r}$ to the normal features $\chi_{h,w}$. The formula is as follows:

(12) $$\chi_{h,w}^{-}=\chi_{h,w}+\epsilon .$$

We use a truncated L1 loss as the loss function for training [26]. L represents the training objective, where ${th}^{+}$ and ${th}^{-}$ are truncation terms to prevent overfitting. The formula is as follows:

(13) $$l_{h,w}^{i}=\max\left(0,{th}^{+}-D\left(q_{h,w}^{i}\right)\right)+\max\left(0,-{th}^{-}+D\left(q_{h,w}^{i-}\right)\right),$$

(14) $$L=\min\sum_{q^{i}\in \chi_{train}}\sum_{h,w}\frac{l_{h,w}^{i}}{H_{0}*W_{0}}.$$

The discriminator D performs discrimination on the features at each position $\left(h,w\right)$ and outputs an anomaly score $s_{h,w}$ for the patch at that position. By performing this evaluation over all patches in the feature map, we obtain an anomaly score map of the same size as the feature map. We use the maximum anomaly score from all patches as the anomaly score $S_{A}$ for the entire image. $S_{A}$ is used to determine whether an image is anomalous, while $s_{h,w}$ is used to assess the anomaly of each individual patch. The formula is as follows:

(15) $$s_{h,w}=-D\left(\chi_{h,w}\right),$$

(16) $$S_{A}=\underset{\left(h,w\right)\in H_{0}\times W_{0}}{\max}s_{h,w}.$$

We upsample the anomaly score map of size $H_{0}\times W_{0}$ to H×W, obtaining an anomaly score output $S_{AL}$ that matches the size of the input image. The score at each position in $S_{AL}$ represents the anomaly score of the corresponding pixel. The formula is as follows:

(17) $$S_{AL}=\left\{s_{h,w}|(h,w)\in H\times W\right\}.$$

## 4. Experiments and Results

### 4.1. Datasets

**Figure 6:**
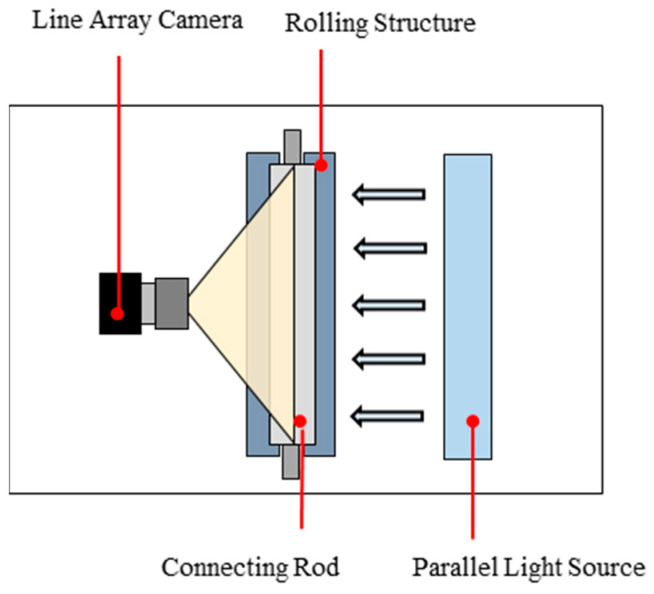
We designed a surface image acquisition system for the connecting rod, which primarily includes a line array camera, a rolling structure, and a parallel light source. The surface image of the connecting rod is captured by rotating it through one full rotation using the rolling structure.

The dataset used in this study consists of surface images of automotive connecting rods, collected from an industrial production line. Since the automotive connecting rods have cylindrical surfaces, their surface images cannot be captured using conventional area array cameras. Therefore, we designed a specialized imaging system for the connecting rods. The system rotates the connecting rod on a roller and uses a Keyence line scan camera (CA-HL08MX) to scan the surface, thereby obtaining an unfolded map of the surface. To ensure consistent image brightness, a parallel light source is employed for illumination. The specific configuration of the system and the placement of the equipment are shown in Figure 6.

**Figure 7:**
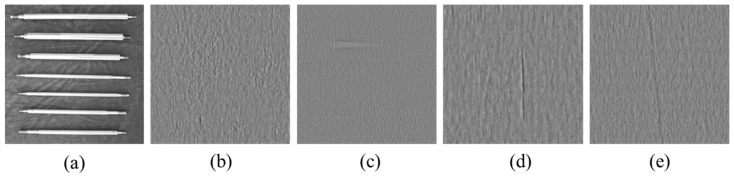
Examples of connecting rod images: (**a**) shows the full image; (**b**–**e**) display cropped local patches.

In this study, data were collected for 100 automotive connecting rod samples, resulting in a total of 400 surface images with a resolution of 6000 × 8000 pixels (four images per connecting rod). To facilitate network training, all full-sized images were cropped to a resolution of 1024 × 1024 pixels. The dataset construction is based on the cropped images. As shown in Figure 7, Figure 7a shows the complete connecting rods, and Figure 7b–e represent cropped patches of the images. Due to the extreme rarity of surface defect samples, only 150 defect-containing images were collected. To balance the dataset, 150 normal surface images were randomly selected to form the test set. Considering the computational resource demands of unsupervised learning, we aimed to minimize the data size for training. As a result, 200 normal images were used as the training set. This dataset is named CRS (connecting rod surface image dataset), consisting of a total of 500 images, where the training set includes 200 normal images, and the test set contains 150 normal images and 150 anomalous images. The dataset is annotated using Labelme. The anomaly regions are annotated using a binary classification segmentation method.

Additionally, we conducted generalization experiments on the Kolektor Surface-Defect Dataset 2 (KSDD2) [40]. Since KSDD2 is a supervised learning dataset, to meet the experimental requirements, we randomly selected 200 normal samples from the original training set as the training set, and 100 normal samples and 100 defect samples from the test set to form a new test set.

### 4.2. Experimental Configuration and Experimental Details

The experiments in this paper were conducted on an Ubuntu 20.04 system, with an Intel i9–13900K CPU and an Nvidia GeForce RTX 4090 GPU, as detailed in Table 2. In the experiments, all input images are uniformly resized to 256 × 256 pixels. The patch size k for the patch-based DCT transformation is set to 4. A pre-trained WideResNet50 was used as the backbone network, with the first, second, and third layers serving as spatial feature extractors. The reconstruction feature scale was unified to 64 × 64, and the feature selection dimension r was set to 1536. The Adam optimizer was used, with the learning rate set to 0.0001 and weight decay set to 0.00001. The number of training epochs for each dataset was set to 100, and the batch size was 4. 

### 4.3. Results of the CRS Dataset

To validate the effectiveness and superiority of the proposed method, we compared it with several representative methods in the anomaly detection field, including PaDiM [20], PatchCore [21], MemSeg [27], and SimpleNet [26]. To assess the performance of these methods, we used the AUROC (area under the receiver operating characteristic curve) and F1 score for both classification and segmentation tasks as evaluation metrics. The calculation formulas are as follows:

(18) $$\mathrm{TPR}(\mathrm{Recall})=\frac{TP}{TP+FN},$$

(19) $$FPR=\frac{FP}{TN+FP},$$

(20) $$Precision=\frac{TP}{TP+FP},$$

(21) $$AUROC=\int_{0}^{1}TPR\left({FPR}^{-1}\left(x\right)\right)dx,$$

(22) $$F1=\frac{2\times Recall\times Precision}{Recall+Precision}.$$

Here, TP represents the anomalous samples correctly classified as anomalies, and FP represents the normal samples incorrectly classified as anomalies. For image-level, the statistics are calculated on a per-image basis, while for pixel-level, the results are computed on a per-pixel basis. The experimental results for the connecting rod are shown in Table 3. We compare the proposed method, FFNet, with several mainstream methods in terms of image classification AUROC, F1 score, as well as pixel segmentation AUROC and F1 score. Our method achieved the best results in both image AUROC and pixel AUROC, with scores of 99.4% and 95.4%, respectively. Additionally, the F1 scores for both image classification and pixel segmentation were the highest among all algorithms, reaching 98.4% and 31.8%, respectively.

**Figure 8:**
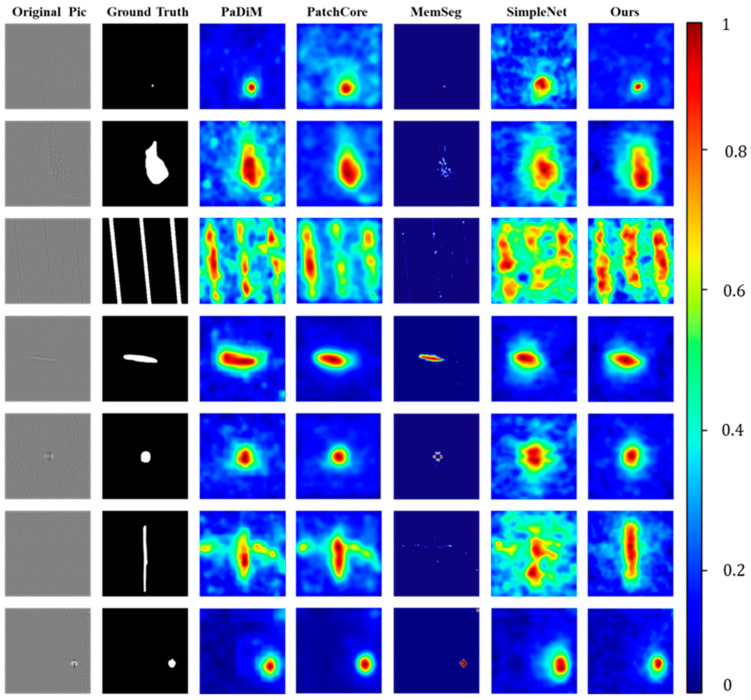
The experimental results on the CRS dataset are visualized as heatmaps. Anomalous outputs are represented in these heatmaps, where higher anomaly scores are more prominently displayed, indicating regions with more significant abnormalities.

As shown in Figure 8, our method demonstrates a clear visual advantage over other methods, particularly excelling in handling samples with mild defects. Compared to SimpleNet, which also uses WideResNet as the backbone, our method performs better in detecting shallow defects that are easily obscured by surface metal textures. This advantage is attributed to the incorporation of frequency domain features, which enhance our method’s ability to perceive and distinguish between textures and defects.

In the segmentation task, although MemSeg excels in detecting obvious defects by adding a segmentation subnet at the end of the network; this design leads to noticeable false negatives in samples with less prominent defect features. As shown in Figure 8, the defect in the fourth sample is more prominent, and the segmentation performance of MemSeg is relatively good. However, the performance on the third and sixth samples is noticeably worse. In industrial applications, the risk of false negatives often has low tolerance. Therefore, our method offers a stronger advantage over these classic anomaly detection methods, particularly in industrial scenarios where high precision is required, ensuring more stable and reliable performance.

**Figure 9:**
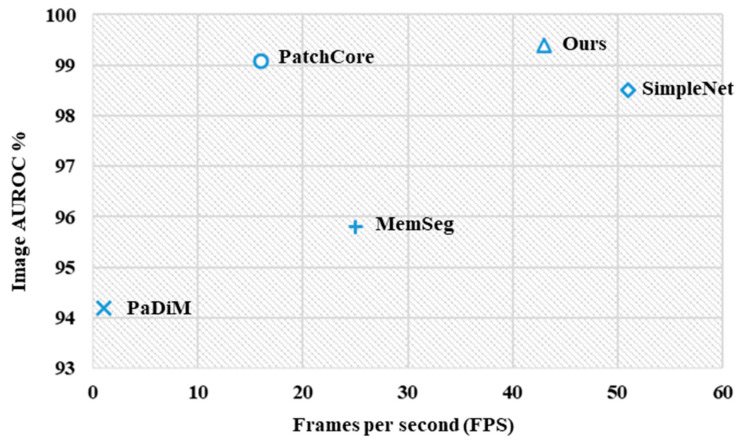
Inference speed versus Image AUROC on CRS.

Additionally, we conducted a comparative analysis of inference speeds across different algorithms. As shown in Figure 9, the *y*-axis represents I-AUROC while the *x*-axis denotes FPS. In this visualization, positions further to the right indicate faster inference speeds (higher FPS values), whereas positions higher up correspond to superior detection performance (elevated Image AUROC). Our method achieves the best detection performance (highest I-AUROC) while closely following SimpleNet in inference speed (second highest FPS). As evidenced by its positioning in the upper-right quadrant of Figure 9, the proposed approach demonstrates an optimal balance between detection efficacy and computational efficiency. Despite the incorporation of additional frequency domain features, our method maintains competitive inference speeds through the optimized architecture of the RFS module.

### 4.4. Results of the KSDD2 Dataset

**Figure 10:**
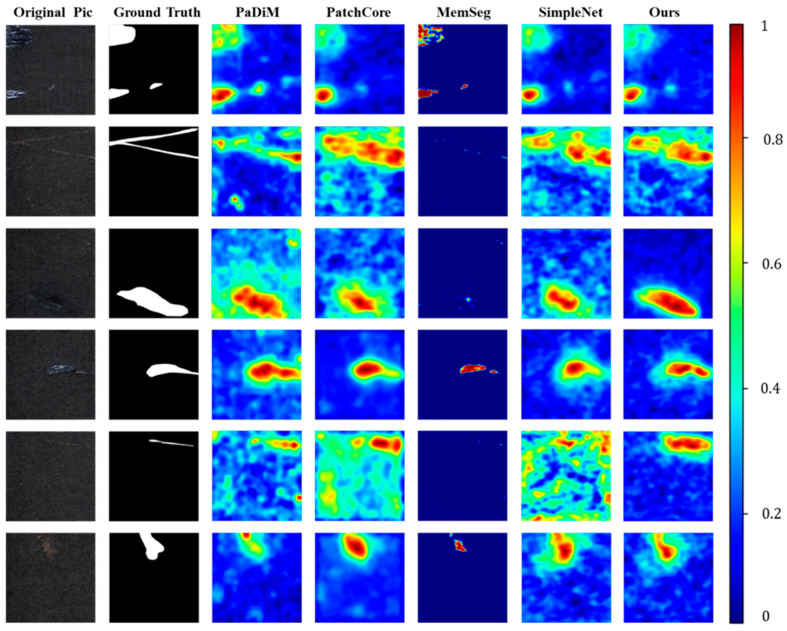
KSDD2 dataset visualization results.

The results are shown in Table 4. On the KSDD2 dataset, our method still achieved the best performance. In terms of image-level AUROC and F1 scores, we reached 96.9% and 93.6%, the highest values among all methods. At the pixel level, our method achieved AUROC and F1 scores of 97.6% and 52.8%, respectively. Although our Pixel F1 score is slightly lower than that of MemSeg, we rank first in all other metrics, especially in Image AUROC, where we outperform the second-best PatchCore by 1.7%. As shown in Figure 10, our method still demonstrates significant visual advantages, particularly excelling in detecting smaller defects. Although MemSeg slightly outperforms our method in Pixel F1 score, its introduction of a segmentation subnet at the end of the network leads to more severe false negatives. Considering all the metrics and false negative situations, our method shows stronger generalization capability compared to MemSeg. This is particularly valuable in practical industrial applications, where it holds great potential for handling various defect detection tasks.

### 4.5. Ablation Experiments

#### 4.5.1. Patch Size for the DCT Transform on the Effectiveness of Detection

As shown in Table 5, we conducted experiments with different patch division sizes. The results indicate that the choice of patch size significantly affects the model’s detection performance. The Patch-based DCT transform demonstrates a clear optimal solution between local and global contexts, with the best model performance achieved when k=4.

#### 4.5.2. Ablation Study on Modules of FFNet

To validate the effectiveness of each component in the algorithm, we conducted a series of ablation experiments on CRS. As shown in Table 6, to ensure consistent intermediate feature dimensions across all positions in the network, we replaced SAFR with concatenation and used the random dimensionality reduction (RDR) [20] instead of RFS.

The following conclusions can be drawn. Without adding any modules, simply incorporating frequency domain information leads to improvements in both Image AUROC and Pixel AUROC compared to the baseline. This indicates that frequency domain features complement spatial domain features, thereby enhancing anomaly perception. After the frequency domain features are enhanced through the FFE module, they are better at representing the differences between anomalous and normal features than the fixed DCT-derived frequency domain features. Furthermore, after using SAFR to reconstruct the frequency and spatial features, the detection performance improved even further. Regardless of whether frequency domain features were added, RFS significantly contributed to improving detection performance.

Overall, these modules have a substantial impact on the model’s detection performance. With the combined effect of these modules, the model’s performance in both image-level and pixel-level tasks improved significantly: Image AUROC and Image F1 increased by 1.3% and 0.8%, respectively, while Pixel AUROC and Pixel F1 improved by 1.7% and 6.5%.

## 5. Conclusions

In this paper, we propose FFNet, a novel unsupervised anomaly detection method based on frequency domain feature fusion, capable of accurately identifying and segmenting surface defects in metals. Our method primarily consists of three core components: the FFE, responsible for extracting and enhancing frequency domain features; the SAFR, responsible for fusing spatial and frequency domain features; and the RFS, responsible for filtering the reconstructed features. These components work together to introduce frequency domain features and effectively integrate them with spatial domain features, thereby enhancing the model’s ability to perceive subtle anomalies while controlling redundant features.

FFNet achieved image-level AUROC and F1 of 99.4% and 93.6%, respectively, and pixel-level AUROC and F1 of 96.9% and 97.6% on the CRS dataset. Compared to other unsupervised anomaly detection algorithms, FFNet demonstrated the best detection performance. We also conducted generalization experiments on the KSDD2 dataset, achieving similarly advanced results, proving the strong generalization ability and robustness of our method. FFnet achieves 43 FPS on an RTX 4090 in terms of inference speed, which is second only to that of the fastest SimpleNet. This indicates that our method remains highly competitive and holds substantial promise for practical industrial applications.

Overall, FFNet has shown excellent detection capability in metal surface defect detection, providing important insights and experiences for future research in this field. It should be noted that our method currently exhibits certain limitations. While FFNet prioritizes distinguishing anomalous regions from normal areas, it achieves suboptimal precision in boundary delineation, particularly for defects with ambiguous edges. Additionally, the framework requires a substantial number of normal samples during training. In future work, we will address these limitations through architectural refinements to enhance boundary localization accuracy and explore few-shot learning paradigms to reduce dependency on large-scale datasets. Next, we also aim to further improve the detection of small defects and extend our algorithm to defect detection tasks for industrial products beyond metals.

## Figures and Tables

**Table 1:** The sizes of the input and output features in the STM module.

Input Feature	Size	Output Feature	Size
$f_{1}^{spac}$	$\frac{H}{4}\times \frac{H}{4}\times 256$	$X_{1}^{spac}$	$H_{0}\times W_{0}\times 256$
$f_{2}^{spac}$	$\frac{H}{8}\times \frac{H}{8}\times 512$	$X_{2}^{spac}$	$H_{0}\times W_{0}\times 512$
$f_{3}^{spac}$	$\frac{H}{16}\times \frac{H}{16}\times 1024$	$X_{3}^{spac}$	$H_{0}\times W_{0}\times 1024$
$f^{freq}$	$\frac{H}{k}\times \frac{H}{k}\times k^{2}$	$X^{freq}$	$H_{0}\times W_{0}\times k^{2}$

**Table 2:** Experiment environment parameters.

Hardware or Software	Version
CPU	Intel 13900K
GPU	RTX 4090
RAM	64G
Operating system	Ubuntu 20.04
CUDA version	12.0

**Table 3:** Comparison results on CRS. Anomaly detection and localization performance are measured based on Image AUROC (%), Image F1 (%), Pixel AUROC (%) and Pixel F1 (%). Additionally, we calculated the execution efficiency of the algorithm, expressed in FPS.

	Image AUROC	Image F1	Pixel AUROC	Pixel F1	FPS
PaDiM	94.2	93.6	91.5	20	1
PatchCore	99.1	97.8	93.6	28	16
MemSeg	95.8	94.2	92.1	22.6	25
SimpleNet	98.5	97.5	87.4	21.5	**51**
**Ours**	**99.4**	**98.4**	**95.4**	**31.8**	43

**Table 4:** Comparison results on KSDD2. Anomaly detection and localization performance are measured based on Image AUROC (%), Image F1 (%), Pixel AUROC (%) and Pixel F1 (%).

	Image AUROC	Image F1	Pixel AUROC	Pixel F1
PaDiM	92.5	89.3	97.1	49.2
PatchCore	95.2	91.3	97.5	50.1
MemSeg	93.3	88.7	93.2	**53.8**
SimpleNet	94.9	92.9	96.8	46.1
**Ours**	**96.9**	**93.6**	**97.6**	52.8

**Table 5:** The impact of different patch division sizes k on detection performance. Anomaly detection and localization performance are measured based on Image AUROC [%], Image F1 [%], Pixel AUROC [%], and Pixel F1 [%].

	Image AUROC	Image F1	Pixel AUROC	Pixel F1
k = 2	98.5	96.1	93.2	**32.2**
k = 4	**99.4**	**98.4**	**93.6**	31.8
k = 8	99.2	98.3	92.4	28.1
k = 16	97.8	96.9	91.5	25.6

**Table 6:** Ablation study of different modules on CRS. Anomaly detection and localization performance are measured based on Image AUROC (%), Image F1 (%), Pixel AUROC (%), and Pixel F1 (%).

Models		I-AUROC	Image-F1	P-AUROC	P-F1
Without frequency	-	98.1	97.6	91.9	25.3
	+RFS	98.6	98.3	92.5	27.6
With frequency	-	98.4	97.4	92.0	24.5
	+FFE	98.6	97.9	93.1	26.4
	+FFE+SAFR	99.1	98.3	93.5	28.9
	+FFE+SAFR+RFS	**99.4**	**98.4**	**93.6**	**31.8**

## Data Availability

Data available on request from the authors.
